# A Comparison of Patient-Reported Outcomes Following Consent for Genetic Testing Using an Oncologist- or Genetic Counselor-Mediated Model of Care

**DOI:** 10.3390/curroncol28020138

**Published:** 2021-04-08

**Authors:** Jeanna M. McCuaig, Emily Thain, Janet Malcolmson, Sareh Keshavarzi, Susan Randall Armel, Raymond H. Kim

**Affiliations:** 1 Familial Cancer Clinic, Princess Margaret Cancer Centre, 610 University Avenue, Toronto, ON M5G 2M9, Canada; Emily.Thain@uhn.ca (E.T.); Janet.Malcolmson@uhn.ca (J.M.); Susan.Randall@uhn.ca (S.R.A.); Raymond.Kim@uhn.ca (R.H.K.); 2 Department of Molecular Genetics, University of Toronto, 27 King’s College Circle, Toronto, ON M5S 1A8, Canada; 3 Department of Biostatistics, Princess Margaret Cancer Centre, 610 University Avenue, Toronto, ON M5G 2M9, Canada; Sareh.Keshavarzi@uhnresearch.ca; 4 Department of Medical Oncology, Princess Margaret Cancer Centre, 610 University Avenue, Toronto, ON M5G 2M9, Canada; 5 Department of Medicine, University of Toronto, 27 King’s College Circle, Toronto, ON M5S 1A8, Canada

**Keywords:** genetic testing, genetic counseling, mainstreaming, service delivery model, hereditary cancer, breast cancer, ovarian cancer

## Abstract

**Simple Summary:**

Genetic testing for hereditary cancer risk is usually arranged by a genetic counselor after talking about possible risks and benefits. To increase access to genetic testing, oncologists have started to order genetic testing. This survey study compared patient outcomes following genetic testing ordered by a genetic counselor or an oncologist. Genetic counselor-mediated genetic testing was associated with higher patient knowledge, as well as higher experience and understanding of genetic testing. Differences were noted in the type of psychological concerns reported, with individuals having genetic counselor-mediated testing being more likely to express concerns about having a hereditary cancer predisposition and those having oncologist-mediated testing more likely to express concerns regarding general emotions. Overall, oncologist-mediated genetic testing appears to provide a streamlined alternative to genetic testing; however, all individuals may benefit from post-test genetic counseling to address any knowledge gaps and provide additional psychological support.

**Abstract:**

This study compares knowledge, experience and understanding of genetic testing, and psychological outcomes among breast and ovarian cancer patients undergoing multi-gene panel testing via genetic counselor-mediated (GMT) or oncologist-mediated (OMT) testing models. A pragmatic, prospective survey of breast and ovarian cancer patients pursuing genetic testing between January 2017 and August 2019 was conducted at the Princess Margaret Cancer Centre in Toronto, Canada. A total of 120 (80 GMT; 40 OMT) individuals completed a survey administered one week following consent to genetic testing. Compared to OMT, the GMT cohort had higher median knowledge (8 vs. 9; *p* = 0.025) and experience/understanding scores (8.5 vs. 10; *p* < 0.001) at the time of genetic testing. Significant differences were noted in the potential psychological concerns experienced, with individuals in the GMT cohort more likely to screen positive in the hereditary predisposition domain of the Psychosocial Aspects of Hereditary Cancer tool (55% vs. 27.5%; *p* = 0.005), and individuals in the OMT cohort more likely to screen positive in the general emotions domain (65.0% vs. 38.8%; *p* = 0.007). The results of this study suggest that OMT can be implemented to streamline genetic testing; however, post-test genetic counseling should remain available to all individuals undergoing genetic testing, to ensure any psychologic concerns are addressed and that individuals have a clear understanding of relevant implications and limitations of their test results.

## 1. Introduction

In the current era of precision medicine, genetic testing has become integral to the care of cancer patients. Among breast and ovarian cancer patients, genetic testing results may alter surgical management [1] or systemic therapy [2], respectively; therefore, a subset of patients may require genetic testing at the time of diagnosis, or shortly thereafter. In addition to this potential treatment-focused urgency of genetic test results, the number of requests for hereditary cancer genetic testing has risen in recent years [3,4]. With a concurrent shortage of clinical genetic counselors to facilitate genetic testing [5], many institutions lack the workforce required to meet increasing demands. In the province of Ontario, referrals for cancer genetic services almost tripled between 2007 and 2016, resulting in an unmet need for 34.7 additional genetic counselors to provide patient care [6]. Consequently, new models of genetic testing are being implemented, where genetic testing is ordered with minimal involvement of genetic professionals [7,8,9,10]. 

In the traditional, genetic counselor-mediated testing model (GMT), individuals are referred to a clinical genetics service, with testing coordinated by a genetic counselor over two visits (pre-test and post-test) [11]. GMT is associated with positive patient outcomes, including increased knowledge, improved accuracy of perceived risk, decreased anxiety, and high patient satisfaction [12]. By comparison, in an oncologist-mediated testing model (OMT), genetic testing is initiated by the oncology team, with involvement of genetics professionals only at the time of results disclosure [10]. At the Princess Margaret Cancer Centre (PM) in Toronto, oncologists are able to facilitate genetic testing for select breast and ovarian cancer patients who are eligible for publicly funded genetic testing based on their cancer diagnosis alone (i.e., irrespective of family history). In this model, participating oncologists discuss genetic testing with eligible patients during a routine oncology visit and provide patients with an information pamphlet about genetic testing. Patients who consent to genetic testing are scheduled a single appointment with a genetic counselor for results disclosure.

Colloquially known as ‘mainstreaming’, the OMT model has been implemented in centers around the world, with many groups reporting decreased time to genetic test results, improved cost-effectiveness, and high levels of patient satisfaction [10,13,14,15,16,17,18,19]. Despite the potential patient impact of genetic testing, there is limited data regarding patient reported outcomes following OMT. Two recent studies have compared OMT and GMT models, reporting no significant differences in patient reported knowledge, satisfaction, or psychological functioning following results disclosure [17,19]. While these results are promising, there remains an absence of patient outcome data following pre-test discussions and consent to genetic testing via OMT.

The aim of the current study is to compare the knowledge, experience and understanding of genetic testing, and psychological concerns reported among breast and ovarian cancer patients pursuing genetic testing using GMT or OMT models of care. By evaluating outcomes following consent to genetic testing, we hope to gain a better understanding of where additional support may be required with the expanded use of OMT. 

## 2. Results

### 2.1. Study Population

**Figure 1:**
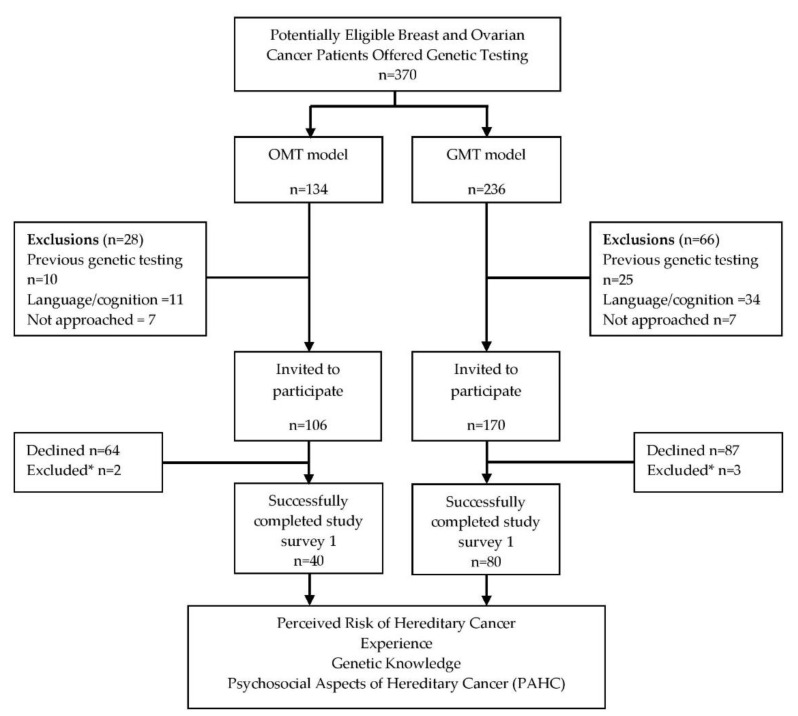
Study Design and Participation. OMT: Oncologist-mediated Genetic Testing; GMT: Genetic Counselor-mediated Genetic Testing. * five individuals (two OMT and three GMT) completed survey 1 after receiving their genetic test results and were excluded from analysis.

A total of 370 breast and ovarian cancer patients were identified and 276 (74.6%) were invited to participate in the study; 83/170 (48.8%) of individuals in the GMT cohort and 42/106 (39.6%) in the OMT cohort consented to participate. Five individuals completed the first study survey after receiving their genetic testing results and were excluded from analysis, providing a total 120 study participants (80 GMT; 40 OMT) (Figure 1). Participants comprised a homogenous group of highly educated females, with primarily (61.7%) Caucasian ancestry. The median age at diagnosis was 57 years; the majority (72.3%) of participants had a diagnosis of ovarian cancer and 62.2% reported a family history of breast/ovarian cancer. There were no significant differences among demographic variables between the two groups (Table 1). 

### 2.2. Survey Responses Following Consent to Genetic Testing

#### 2.2.1. Experience and Understanding of Genetic Testing

Overall, participants agreed with a median of 10 items on the patient experience and understanding scale. The GMT cohort had a significantly higher median score (10 versus 8.5 in OMT cohort; *p* < 0.001) (Table 2). On univariable analysis, individuals in the OMT cohort agreed with significantly fewer items on this scale (β = −1.73; *p* < 0.001) (Table S1). When evaluating the specific items (Table 3), the OMT cohort was significantly less likely to report that the information they received about genetic testing was clear and helpful (*p* = 0.01) or given in a way they could understand (*p* = 0.01). They were also less likely to report the information provided helped them understand how genetic testing might impact themselves (*p* = 0.003) or their family (*p* = 0.003). Furthermore, individuals in the OMT cohort were less likely to report understanding the possible results of genetic testing (*p* = 0.001) and only 66.7% (versus 91.3% in GMT cohort; *p* = 0.001) were aware that they could contact a genetic counselor if they had questions before deciding to have genetic testing. When asked if they felt the process of having genetic testing worked well, the majority of participants agreed; however, significantly fewer of those who had OMT agreed (*p* = 0.02).

#### 2.2.2. Genetic Testing Knowledge and Perceived Hereditary Cancer Risk

Following consent to genetic testing, individuals in the GMT cohort had a higher knowledge scores compared to those in the OMT cohort, with a median of nine and eight correct responses, respectively (*p* = 0.025) (Table 2). Univariable analysis was completed to identify factors associated with knowledge scores (Table S1); OMT was associated with a significantly lower number of correct responses (β = −1.03; *p* = 0.009). Regarding specific items (Table 4), both groups scored lowest on items related to the possible results of genetic testing and male inheritance; however, the OMT group scored significantly lower on these items (40.0% versus 63.3% in GMT; *p* = 0.02 and 20.0% versus 53.2% in GMT; *p* = 0.001, respectively).

Among the total study cohort, the reported perceived risk of hereditary cancer ranged from 0% to 100%, with a median score of 30%. The GMT cohort had a higher median perceived risk (40%) compared to OMT (22.5%) (*p* = 0.29) (Table 2). While there was no significant difference between OMT and GMT groups, univariable analysis found that a positive family history of breast/ovarian cancer was associated with a 20.3% higher perceived risk, compared to no reported family history (*p* < 0.001) (Table S1). 

### 2.3. Psychosocial Aspects of Hereditary Cancer (PAHC)

**Figure 2:**
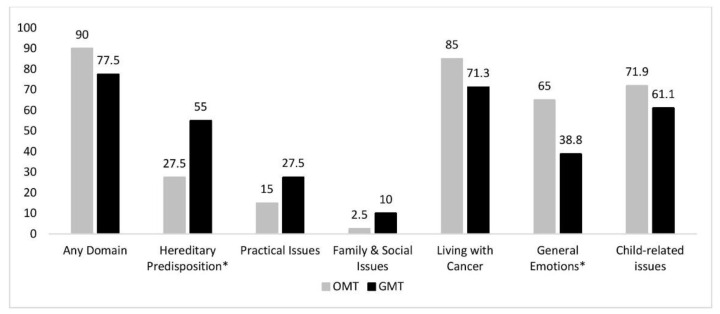
Proportion of individuals who screened positive on Psychosocial Aspects of Hereditary Cancer. GMT: Genetic counselor-mediated genetic testing; OMT: oncologist-mediated genetic testing. * OMT and GMT differed significantly in the proportion who screened positive on the Hereditary Predisposition (*p* = 0.005) and General Emotions (*p* = 0.007) domains of the PAHC tool.

The majority of individuals in both cohorts screened positive in at least one domain of the PAHC tool (i.e., scored 3 or 4 on any item of a particular domain) (Table 2; Figure 2). Compared to OMT, a significantly higher proportion of individuals in the GMT cohort screened positive on the “hereditary predisposition” domain (55.0% vs. 27.5%; *p* = 0.005), whereas a significantly higher proportion of individuals in the OMT cohort screened positive on the “general emotions” domain (*p* = 0.007) (Table 2, Figure 2). These findings were confirmed via univariable modeling (Table S2). OMT remained a significant predictor of screening positive on the “general emotions” domain in a multivariable model which included genetic testing group and age at diagnosis (OR = 1.27; *p* = 0.01) (Table S3). Additional multivariable analyses (Table S3) showed individuals with a diagnosis of ovarian cancer were significantly more likely to screen positive on the “practical issues” domain (OR = 3.71; *p* = 0.04), while increasing age decreased the likelihood of screening positive on this domain (OR = 0.94, *p* = 0.01) as well as the “family and social issues” domain (OR = 0.997, *p* = 0.04).

### 2.4. Survey Responses Following Receipt of Genetic Testing Results

Of the initial 120 participants, 89 (74.2%) completed a second study survey (60 GMT; 29 OMT) one week following disclosure of genetic test results. Given the small number of responses, detailed results are presented in Supplemental Materials (Tables S4–S6). Congruent with data reported by others [17,19], there were no significant differences between OMT and GMT groups with respect to knowledge scores or level of psychological impact of receiving genetic test results. Among 89 individuals who completed both surveys, post-test counseling appeared to improve knowledge scores in the OMT group (β = +0.95; *p* = 0.035). Overall, the majority of individuals (97.7%) were able to correctly recall their test results, and most (94.4%) had discussed their genetic testing results with at least one relative.

## 3. Discussion 

This study provides comparisons of various outcomes reported by individuals with breast or ovarian cancer pursuing genetic testing via GMT or OMT. In our study cohort, GMT appears to result in better knowledge and understanding of genetic testing for hereditary cancer. Differences were also noted in the types of psychological concerns expressed at the time of genetic testing consent, which may be important for oncologists and genetic counselors to consider. Though limited by a low response rate and small sample size, the results of this study reveal potential differences in the level of knowledge and potential psychological impact of genetic testing using GMT or OMT models of care, which can be addressed during post-test counseling. 

Consistent with the high levels of patient satisfaction and acceptability of OMT reported by others [10,13,14,17,19], the overall OMT cohort agreed with most items on our experience and understanding scale (median 8.5 of 10 items) and 74% agreed that the process of having genetic testing worked well. Interestingly, a third of individuals in our OMT cohort were not aware that they could speak to a genetic counselor prior to testing. While this finding may seem concerning, 93% of these individuals, and the OMT cohort overall, reported they had enough information to proceed with genetic testing. Though the option of pre-test counseling may not be obvious to all OMT patients, these results suggest that many breast or ovarian cancer patients may not feel that formal genetic counseling is necessary for most to make a decision about genetic testing. Nevertheless, if the option of meeting with a genetic counselor prior to genetic testing is available, it should be clear so that individuals who do wish to have a more detailed conversation prior to consenting to testing have the opportunity to do so.

When evaluating the information provided to patients at the time of genetic testing, our results suggest that the information provided during OMT was less clear, did not provide as much detail about the implications of genetic testing, and did not obviously delineate the possible results of testing. The OMT group also appeared to be less knowledgeable regarding the potential for male transmission of hereditary breast/ovarian cancer risk, a common misconception that our group has previously described [20]. The written information provided to patients was developed by a team of genetic counselors, geneticists, and oncologists, in conjunction with patient advocacy groups and patient education experts. Though the information pamphlet was piloted with a small group of patients, included information on male inheritance, and contained subsections describing ‘how can genetic testing help me?’, ‘how can genetic testing help my family?’ and ‘what are the possible results of my genetic testing?’, it is not known how many individuals in the OMT group read the information. Furthermore, despite providing key information about genetic testing and example phrases to ordering oncologists, it is unknown what information was discussed with patients when genetic testing was offered. Our study results may serve as a reminder to oncologists offering genetic testing at our institution, and elsewhere, to provide clear and detailed information to patients at the time of testing. For genetic counselors who may see patients for post-test counseling, the results of this study may provide a framework as to what information is most relevant to review during results disclosure.

Typically, GMT involves detailed pre-test genetic counseling and formal risk assessment [11]. While the pre-test education likely explains why our GMT cohort reported a higher median knowledge scores, it may also explain why this cohort reported a higher perceived risk of hereditary cancer (40% vs. 22.5% for OMT) and were significantly more likely to screen positive on the ‘hereditary predisposition’ domain of the PAHC tool (55% vs. 27.5%). In contrast, the OMT cohort was more likely to screen positive on the ‘general emotions’ domain of the PAHC tool (67.5% vs. 50%), which relates to anxiety, depression, and concerns about death, and may reflect prognostic-focused conversations with their oncologist at the time of testing. The high screen positive rate with the PAHC tool overall is consistent with published data [21]; yet, the differing trends in the proportion of participants in each cohort who screened positive in a particular domain provides valuable insight into relevant issues to address during the genetic testing process. For example, individuals in the OMT cohort may require additional emotional support about their diagnosis; they may also benefit from a more detailed discussion of the psychological implications of their genetic testing results as they may not yet have had an opportunity to discuss these issues. In contrast, genetic counselors should also remain cognizant of the potential psychological impact of the detailed pre-test discussions that occur during GMT, and ensure there is ample time to discuss any concerns that may arise.

Though the response to our second study survey was limited, interesting trends were noted. In particular, similar MICRA scores were reported among OMT and GMT cohorts, which is consistent with reports from others [17,19]; yet, individuals with a positive family history of breast/ovarian cancer reported significantly lower positive experience scores following result disclosure (β = −2.72 *p* = 0.022). While genetic testing without pre-test genetic counseling does not seem to increase levels of distress above that related to a cancer diagnosis [7,13], published data of breast cancer patients suggests those with a relevant family history are more likely to expect a positive result and express a lack of closure if a hereditary risk is not identified [22]. In our cohort, individuals with a family history of breast/ovarian cancer reported a 20.3% higher perceived hereditary cancer risk (*p* < 0.001) at the time of genetic testing. Thus, while the risk of significant distress following OMT is likely low, additional post-test support may be of particular benefit to individuals with a family history of cancer, irrespective of results. 

In addition to psychological support, post-test counseling may also serve to fill in the knowledge and understanding gaps noted at the time of genetic testing consent. Again, limited by small numbers, individuals in the OMT cohort who completed both study surveys had improved knowledge scores following post-test genetic counseling (β = +0.95; *p* = 0.035). Congruent with Richardson et al., we did not identify a significant difference in post-test knowledge scores between OMT and GMT cohorts following results disclosure [17]. Irrespective of knowledge scores, most individuals correctly recalled their genetic testing results (97.7%) and had discussed their results with at least one family member in the first week following disclosure (94.4%). It is important to note that post-test counseling was provided to all patients in our study cohort, which may have served to increase accurate test recall and highlight the importance of sharing genetic testing results with biological relatives. Additional studies evaluating outcomes of OMT among cohorts with and without post-test counseling are required to further assess the impact of post-test genetic counseling on patient reported outcomes.

### Limitations

The results of our study should be considered in the context of several limitations. Study results were based on a homogenous sample of highly educated, Caucasian women at a single institution and may not be representative of a more diverse population of individuals. The telephone recruitment strategy employed in this study likely contributed to a relatively low participation rate and small sample size, which may have introduced additional biases in our study cohort and limited our ability to detect small differences between the GMT and OMT groups. Additional multi-center studies evaluating a larger and more diverse patient population may provide additional insights into patient needs. Given the novelty of OMT, we elected to use clinician-developed tools to evaluate participant knowledge and experience with genetic testing; thus, our findings should be interpreted with caution. We also employed a pragmatic approach to the study design, which did not allow us to randomize patients to OMT or GMT groups or control the specific details provided by the clinicians to the participants in the OMT group. While this approach may limit study rigor, it better reflects the real-world experience of cancer patients. Finally, the OMT model used in this study included post-test counseling for all patients, irrespective of results; therefore, results may not reflect patient outcomes in OMT models where post-test counseling is not provided to all patients. 

## 4. Materials and Methods

### 4.1. Study Design

#### 4.1.1. Study Participants and Recruitment

Beginning in January 2017, specific oncologists at the Princess Margaret Cancer Centre (PM) began ordering multi-gene panel testing via OMT. Initially reserved for high-grade serous ovarian cancer, OMT protocols expanded to include select breast cancer indications in August 2018 (criteria outlined in Section 4.1.3). To evaluate differences in knowledge, experience/understanding, and psychological functioning, we conducted a prospective survey study of females diagnosed with breast or ovarian cancer undergoing OMT or GMT at the PM. A pragmatic approach was used, whereby patients were offered genetic testing via OMT or GMT based on existing criteria for OMT and the preferred practice of individual oncologists. Ovarian cancer patients were enrolled from January 2017–August 2019, with the addition of breast cancer patients from August 2018–August 2019. All patients were offered multi-gene panel genetic testing, which included analysis of at least 20 genes relevant to breast/ovarian cancer risk. Patients were excluded for the following reasons: (1) previous genetic testing for hereditary cancer, (2) a relative with a known pathogenic variant in a hereditary cancer gene, or (3) unable to complete study surveys due to language or cognitive barriers. Study eligible patients were asked to complete a survey one week after consenting to genetic testing to obtain demographic information and outcomes of interest. Relevant clinical information (personal/family history of cancer, age at diagnosis, ethnicity, and genetic testing results) was collected from participant medical records. A second study survey was provided to participants one week following results disclosure to measure levels of knowledge and distress.

After consenting to genetic testing, eligible patients were provided with a study information package, which included an introductory letter, a participant information form, and a copy of the first study survey. Approximately 1 week later, potential participants received a follow-up phone call from a member of the study team to obtain informed consent. To reduce any perceived coercion to participate in the study, the clinicians offering genetic testing did not engage in discussions about the study with their patients and clinicians were not informed as to whether their patients had consented to participate in the study. All participants provided informed consent before they participated in the study. The study was conducted in accordance with the Declaration of Helsinki, and the protocol was approved by the Research Ethics Board at the University Health Network (REB 16-6199).

#### 4.1.2. Genetic Counselor-Mediated Genetic Testing

In our GMT model, breast and ovarian cancer patients were referred to the Familial Cancer Clinic at PM for genetic testing. The GMT model was used for breast/ovarian cancer patients who did not meet OMT criteria, or whose oncologist did not facilitate testing directly. All patients were provided personal and family history questionnaires to complete in advance of their pre-test genetic counseling appointment. During this appointment, one of six genetic counselors reviewed each patient’s personal and family history of cancer to determine their eligibility for genetic testing. When appropriate, the benefits and limitations of genetic testing were discussed, and genetic testing was offered. For consenting patients, bloodwork was arranged and a post-test appointment with a genetic counselor was scheduled to review test results and provide appropriate recommendations for cancer screening. 

#### 4.1.3. Oncologist-Mediated Genetic Testing

In the OMT model, oncologists facilitated genetic testing for patients with one of the following diagnoses: (1) high-grade serous ovarian cancer; (2) breast cancer diagnosed at or under 35 years of age; (3) triple negative breast cancer diagnosed at or under 60 years of age; (4) bilateral breast cancer, with the first case diagnosed at or under 50 years of age; or (5) breast and ovarian cancer (any histology). A total of four gynecologic oncologists and 15 breast oncologists ordered genetic testing for at least one breast or ovarian cancer patient during the study timeframe. When offering testing, oncologists provided patients with an information pamphlet (available at: https://www.uhn.ca/PatientsFamilies/Health_Information/Health_Topics/Documents/Learn_about_Genetic_Testing.pdf; accessed on 22 February 2021) and initiated a referral to the Familial Cancer Clinic. All patients were pre-booked a single appointment with a genetic counselor to disclose their genetic test results. Recommendations for cancer screening were provided based on the reported family history and available genetic testing results. 

### 4.2. Study Measurements

#### 4.2.1. Experience and Understanding of Genetic Testing 

Patient experience with the consent process and their understanding of genetic testing was evaluated using a novel 10-item tool, with agree, disagree, and unsure response options (Table 3) (KR = 0.83). Though developed internally, questions were based on the patient feedback questionnaires employed by the Mainstreaming Genetic Testing Program [10].

#### 4.2.2. Knowledge and Perceived Hereditary Cancer Risk

Genetic knowledge was evaluated using a novel 11-item knowledge tool with yes, no, and unsure response options (Table 4) (KR = 0.57). KR20 improved to 0.66 after removing items 4, 7, and 9; however, these items were felt to be clinically relevant and were retained for study analysis. Perceived hereditary cancer risk was evaluated using a single ranking question (0–100%). 

#### 4.2.3. Modified Psychosocial Aspects of Hereditary Cancer (PAHC) Tool

Designed to identify the range of psychological issues related to cancer genetic counseling and testing, the Psychosocial Aspects of Hereditary Cancer (PAHC) tool is a 26-item questionnaire with 4-point scaling responses divided into six problem domains that may exist at the time of testing. These domains include: hereditary predisposition, family issues, practical & social issues, living with cancer, general emotions, and child-related issue [23]. A score of 3 or 4 on any item within a given domain is considered a positive screen, suggesting additional psychological assessment is warranted. A total of 20 items were scored in our study population, with reliability scores for individual domains ranging from α = 0.60–0.89 (Table S7). Items that were not deemed relevant to ovarian cancer patients (i.e., worry about decisions to complete their family) were not included. At the request of the institution’s research ethics board, some items were added to provide positive aspects to genetic testing; however, these items were not scored.

#### 4.2.4. Items Included in Survey Administered Following Results Disclosure

In addition to the knowledge survey described in 4.2.2, the Multidimensional Impact of Cancer Risk Assessment (MICRA) questionnaire was administered one week after disclosure of genetic testing results. Developed to measure the psychological impact of receiving cancer genetic test results, the MICRA questionnaire is superior to other validated distress scales in distinguishing between individuals receiving positive *BRCA1/2* results and those receiving other genetic testing results (i.e., negative or inconclusive) [24,25]. It is comprised of 21 four-point response items to provide an overall distress score (α = 0.86), as well as subscales with scores for genetic testing-related distress (six items α = 0.90), uncertainty (nine items α = 0.82), and positive experiences (four items: α = 0.69). The participants were also asked to recall the result of their genetic testing, which was compared to the result recorded in their medical record, and with whom they had shared their genetic test results.

### 4.3. Statistical Analysis

Descriptive statistics were used to summarize demographic information and participant survey responses. Bivariate analyses comparing GMT and OMT cohorts were completed using Mann–Whitney U-tests and Pearson’s Chi Square tests for continuous and categorical variables, respectively. Linear and logistic regression models were completed to determine associated factors with continuous and categorical outcomes, as appropriate. Genetic testing group (GMT vs. OMT), cancer type (breast vs. ovary), age at diagnosis, and family history of breast/ovarian cancer (none vs. any) were included in univariable analysis; additional multivariable analyses were completed for any covariates with *p* < 0.2. Reliability statistics are reported using Cronbach’s α or Kuder–Richardson (KR20) for continuous or dichotomous items, respectively. Statistical analyses were completed using R (R Foundation for Statistical Computing, https://www.R-project.org/; accessed on 14 January 2021) and statistical significance was reported using a two-tailed α = 0.05.

## 5. Conclusions

Overall, the results of this study demonstrate that GMT may provide patients with increased knowledge and understanding of genetic testing at the time of consent. While differences were noted in potential psychological concerns at the time of testing, these can be addressed at the time of results. Overall, OMT appears to be a viable alternative to access genetic testing; however, where possible, post-test counseling should remain available to all patients, to provide additional knowledge and support. This may be particularly relevant for those individuals with a positive family history of cancer, who may have a higher perceived risk of hereditary cancer.

## Figures and Tables

**Table 1:** Comparison of participant demographics in GMT versus OMT cohorts.

Covariate	Full Sample (*n* = 120)	GMT (*n* = 80)	OMT (*n* = 40)	*p*-Value
Marital Status ^a^ (%)				0.35
In a Relationship	10 (8.4)	8 (10.1)	2 (5.0)	
Married/Common-Law	73 (61.3)	45 (57.0)	28 (70.0)	
Single/Widowed	36 (30.3)	26 (32.9)	10 (25.0)	
Education Level ^b^ (%)				0.67
Elementary/Middle S	3 (2.5)	1 (1.3)	2 (5.0)	
High School	18 (15.2)	12 (15.4)	6 (15.0)	
Certificate Program	11 (9.3)	6 (7.7)	5 (12.5)	
College/University	58 (49.3)	40 (51.3)	18 (45.0)	
Post-Graduate	28 (23.7)	19 (24.4)	9 (22.5)	
Diagnosis (%)				1
Breast	33 (27.7)	22 (27.8)	11 (27.5)	
Ovarian ^c^	86 (72.3)	57 (72.2)	29 (72.5)	
Age at diagnosis				0.18
Mean (sd)	57.4 (11.1)	58.4 (11.0)	55.4 (11.3)	
Median (Min,Max)	57 (24,79)	57 (32,79)	54.5 (24,78)	
Family history of BR/OV cancer ^a^ (%)				0.80
No	45 (37.8)	31 (39.2)	14 (35.0)	
Yes	74 (62.2)	48 (61.8)	26 (65.0)	
Ethnicity				0.69
African	1 (0.8)	1 (1.3)	0 (0)	
Ashkenazi Jewish	10 (8.3)	9 (11.3)	1 (2.5)	
Asian	15 (12.5)	11 (13.8)	4 (10.0)	
Caucasian	74 (61.7)	45 (56.3)	29 (72.5)	
East Indian	6 (5.0)	4 (5.0)	2 (5.0)	
Hispanic	2 (1.7)	1 (1.3)	1 (2.5)	
Middle Eastern	3 (2.5)	2 (2.5)	1 (2.5)	
Mixed	2 (1.7)	2 (2.5)	0 (0)	
West Indies	3 (2.5)	2 (2.5)	1 (2.5)	
Missing/Unknown	4 (3.3)	3 (3.8)	1 (2.5)	

GMT: Genetic counselor-mediated genetic testing; OMT: oncologist-mediated genetic testing; BR: breast; OV: ovarian. ^a^ Marital status and family history were missing for one individual in GMT group. ^b^ Education was missing for two individuals in GMT group. ^c^ two individuals with a history of breast and ovarian cancer were included in the ovarian cancer group; diagnosis was unknown for one individual.

**Table 2:** Comparison of survey 1 responses in GMT versus OMT cohorts.

Outcome of Interest	Full Sample	GMT	OMT	*p*-Value
Experience & Understanding				<0.001
Median (Min,Max)	10 (1,10)	10 (3,10)	8.5 (1,10)	
Mean (sd)	8.8 (2.1)	9.4 (1.3)	7.7 (2.7)	
Knowledge Score				0.025
Median (Min,Max)	8 (1,11)	9 (4,11)	8 (1,10)	
Mean (sd)	7.8 (2.1)	8.2 (1.8)	7.1 (2.3)	
Perceived Risk ^a^				0.29
Median (Min,Max)	30 (0,100)	40 (0,100)	22.5 (0,100)	
Mean (sd)	34.6 (29.4)	36.1 (28.7)	31.5 (30.9)	
PAHC-Screen Positive by domain (%)				
Hereditary Predisposition	55 (45.8)	44 (55.0)	11 (27.5)	0.005
Practical Issues ^b^	28 (23.3)	22 (27.5)	6 (15.0)	0.15
Family & Social Issues	9 (7.5)	8 (10.0)	1 (2.5)	0.14
Living with Cancer	91 (75.8)	57 (71.3)	34 (85.0)	0.10
General Emotions	57 (47.5)	31 (38.8)	26 (65.0)	0.007
Child-related issues ^c^	53 (65.4)	30 (61.2)	23 (71.9)	0.33
Any	98 (86.7)	62 (77.5)	36 (90.0)	0.10

GMT: Genetic counselor-mediated genetic testing; OMT: oncologist-mediated genetic testing. Survey 1 Results are reported for 120 participants (80 GMT and 40 OMT); survey 2 results are reported for 89 participants (60 GMT and 29 OMT). ^a^ Responses missing for 13 participants (9 GMT; 4 OMT). ^b^ Response missing for one OMT participant. ^c^ Not applicable for 39 participants (31 GMT; 8 OMT) who did not have biological children.

**Table 3:** Responses to experience and understanding of genetic testing questions.

Thinking about How you Received Information about Genetic Testing, Please Answer the Following ^a^			
Staement Provided	% Agreed		*p*-Value
	GMT	OMT	
a. The information that I was given about genetic testing was clear and helpful.	96.2	75.0	0.01
b. The information was given to me in a way that I could understand.	97.4	77.5	0.01
c. The information helped me understand why I was being offered genetic testing.	96.3	85.0	0.06
d. I knew that I could decide NOT to have genetic testing.	93.8	90.0	0.48
e. The information helped me understand how the result of genetic testing might impact me.	91.3	69.2	0.003
f. The information helped me understand how the result of genetic testing might impact my family.	93.8	71.8	0.003
g. I had enough information to decide whether or not I wanted to have genetic testing.	96.3	92.5	0.39
h. I understand the different types of test results I can receive from my genetic test (positive, negative, inconclusive).	93.8	70.0	0.001
i. I knew that I could contact a genetic counsellor if I had questions before deciding to have genetic testing.	91.3	66.7	0.001
j. Overall, I felt the process of having genetic testing worked well.	91.1	74.4	0.02

GMT: Genetic counselor-mediated genetic testing; OMT: oncologist-mediated genetic testing. ^a^ Participants were given “Agree” “Disagree” and “Unsure” response options; Responses were dichotomized as “Agreed” or “Did Not Agree”.

**Table 4:** Responses to genetic testing knowledge questions.

The Following Questions will Ask You about Hereditary Cancer. Please Answer the Following ^a^			
Statement Provided	% Correct		*p*-Value
	GMT	OMT	
a. All people who have a mutation in a cancer gene will get cancer. (N)	75.0	72.5	0.83
b. A person who has a mutation in a cancer gene has an increased chance to get more than one cancer in their lifetime. (Y)	73.8	55.0	0.06
c. There are only two possible results of a genetic test (i.e., positive or negative). (N)	63.3	40.0	0.02
d. Genetic testing can determine if a cancer is hereditary. (Y)	78.2	78.9	1.00
e. The son of a woman with a mutation in an ovarian cancer gene has a 50% risk of having the mutation. (Y)	53.2	20.0	0.001
f. A genetic test can find 100% of all possible gene mutations. (N)	68.4	59.0	0.41
g. If someone has a mutation in a cancer gene, genetic testing becomes available to their family members. (Y)	72.5	71.8	1.00
h. Some people may feel anxious or guilty during or after genetic counselling and testing. (Y)	83.8	77.5	0.46
i. It is my responsibility to share my test results with my healthcare providers and family members. (Y)	78.8	85.0	0.47
j. For people who have a mutation in a cancer gene, there are medical options to reduce cancer risks. (Y)	81.3	72.5	0.35
k. Women who have a mutation in a cancer gene, only need to share the results with their female family members. (N)	92.4	87.5	0.50

GMT: Genetic counselor-mediated genetic testing; OMT: oncologist-mediated genetic testing. ^a^ Participants were given “Yes” “No” and “Unsure” response options. Correct responses provided: Y = Yes; N = No. Responses were dichotomized as “Correct” or “Not Correct”.

## Data Availability

The data presented in this study are available on request from the corresponding author. The data are not publicly available due to the details included in the study consent form.

## Supplementary Materials

The following are available online at https://www.mdpi.com/article/10.3390/curroncol28020138/s1, Table S1: Univariable analysis of perceived risk and patient experience & understanding, Table S2: Univariable analysis of domains of the PAHC tool, Table S3: Multivariable analysis for Select Domains of the PAHC tool, Table S4: Survey 2 Participant Demographics and Survey Responses, Table S5: Multivariable analysis for changes in knowledge scores, Table S6: Univariable analysis of MICRA questionnaire, Table S7: Modified PAHC Tool.
