# Supplementary material for: A Comparison of Patient-Reported Outcomes Following Consent for Genetic Testing Using an Oncologist- or Genetic Counselor-Mediated Model of Care
Source: Curr Oncol. 2021 Apr 8;28(2):1459–71. doi: 10.3390/curroncol28020138 (PMC8167594; doi:10.3390/curroncol28020138)
Supplement: Supplementary file 1 [file curroncol-28-00138-s001.pdf]

Table S1. Univariable analysis of perceived risk, patient experience & understanding, and knowledge.

**Covariate**	**Experience & Understanding**		**Perceived Risk**		**Knowledge**	
	**β (95% CI)**	**p-value**	**β (95% CI)**	**p-value**	**β (95% CI)**	**p-value**
**Group**		**<0.001**		0.45		**0.009**
GMT	Reference		Reference		Reference	
OMT	−1.73 (−2.44, −1.01)		−4.6(−16.41, 7.21)		−1.03 (−1.79, −0.26)	
**Diagnosis**		0.84		0.42		0.39
Breast	Reference		Reference		Reference	
Ovarian	0.09(−0.74, 0.92)		5.15(−7.46, 17.77)		−0.36 (−1.19, 0.47)	
**Age at diagnosis**	−0.02(−0.05, 0.01)	0.28	−0.02(−0.54, 0.5)	0.93	−0.006 (−0.004, 0.03)	0.72
**Family History**		0.39		**<0.001**		0.92
No	Reference		Reference		Reference	
Yes	−0.34(−1.11, 0.43)		20.31(9.13, 31.48)		−0.04 (−0.81, 0.73)	

Total sample size of n=120

Table S2. Univariable analysis of domains of the PAHC tool.

Covariate	Hereditary Predisposition		Practical & Social Issues		Family Issues		Living with Cancer		General Emotions		Child-related Issues		Any	
	**β (95% CI)**	***p*-value**	**β (95% CI)**	***p*-value**	**β (95% CI)**	***p*-value**	**β (95% CI)**	***p*-value**	**β (95% CI)**	***p*-value**	**β (95% CI)**	***p*-value**	**β (95% CI)**	***p*-value**
**Group**		**0.005**		0.14		0.17		0.1		**0.008**		0.33		0.1
GMT	Reference		Reference		Reference		Reference		Reference		Reference		Reference	
OMT	0.31 (0.14, 0.71)		−0.12 (−0.28, 0.04)		0.23 (0.03, 1.91)		2.29 (0.85, 6.18)		2.94 (1.33,6.47)		0.11 (−0.11, 0.32)		2.61 (0.82, 8.32)	
**Diagnosis**		0.92		0.17		0.7		0.55		0.37		0.41		0.66
Breast	Reference		Reference		Reference		Reference		Reference		Reference		Reference	
Ovarian	1.04 (0.47, 2.34)		0.12 (−0.05, 0.29)		0.75 (0.18, 3.19)		1.32 (0.53, 3.32)		0.69 (0.31,1.55)		0.1 (−0.14,0.33)		0.78 (0.262.34)	
**Age at diagnosis**	1.00 (0.96, 1.03)	0.79	0.97 (0.93, 1.01)	0.1	0.95 (0.89, 1.01)	0.08	0.98 (0.94, 1.02)	0.98	0.97 0.94,1.0)	0.06	−0.009 (−0.02, 9e-03)	0.07	0.96 (0.91, 1.0)	0.05
**Family History**		0.65		0.14		0.77		0.85		0.36		0.59		0.31
No	Reference		Reference		Reference		Reference		Reference		Reference		Reference	
Yes	0.84 (0.4, 1.77)		−0.12 (−0.28, 0.04)		1.24 (0.29, 5.21)		1.08 (0.45, 2.59)		0.7 (0.33,1.48)		−0.06 (0.28, 0.16)		1.64 (0.63, 4.23)	

Total sample size of n=120

Table S3. Multivariable analysis for Select Domains of the PAHC tool.

**Covariate**	**OR**	**95% CI**	***p*-value**
**PRACTICAL & SOCIAL ISSUES DOMAIN**			
**Group**			0.08
GMT	Reference		
OMT	0.39	0.13, 1.12	
**Diagnosis**			**0.04**
Breast	Reference		
Ovarian	3.71	1.04, 13.25	
**Age at diagnosis**	0.94	0.9, 0.99	**0.01**
**Family history**			0.26
No	Reference		
Yes	0.59	0.24, 1.48	
**FAMILY ISSUES DOMAIN**			
**Group**			0.08
GMT	Reference		
OMT	0.91	0.83, 1.01	
**Age at diagnosis**	0.997	0.995, 0.999	**0.04**
**GENERAL EMOTIONS DOMAIN**			
**Group**			**0.01**
GMT	Reference		
OMT	1.27	1.05, 1.53	
**Age at diagnosis**	0.99	0.99, 1.0	0.11
**ANY DOMAIN**			
**Group**			0.19
GMT	Reference		
OMT	1.1	0.95, 1.27	
**Age at diagnosis**	0.99	0.99, 1.0	0.07

Total sample size of n=120

Table S4. Survey 2 Participant Demographics and Survey Responses.

Covariate	Full Sample (n = 89)	GMT (n = 60)	OMT (n = 29)	p-value
**Marital Status[a] (%)**				0.67
In a Relationship	6 (6.8)	5 (8.5)	1 (3.5)	
Married/Common-Law	52(59.1)	33 (55.9)	19 (65.5)	
Single/Widowed	30 (34.1)	21 (35.6)	9 (31.0)	
**Education Level[a] (%)**				0.97
Elementary/Middle S	3 (3.4)	1 (1.7)	2 (6.9)	
High School	12 (13.6)	8 (13.6)	4 (13.8)	
Certificate Program	6 (6.8)	5 (8.5)	1 (3.4)	
College/University	46 (52.3)	32 (54.2)	14 (48.3)	
Post-Graduate	21 (23.9)	13 (22.0)	8 (27.6)	
**Diagnosis (%)**				0.94
Breast	25 (28.1)	17 (28.3)	8 (27.6)	
Ovarian	64 (71.9)	43 (71.7)	21 (72.4)	
**Age at diagnosis**				0.35
Mean (sd)	57.8 (11.2)	58.7 (10.6)	55.9 (12.0)	
Median (Min,Max)	57 (24,78)	57.5 (33, 78)	56. (24,78)	
**Family history of BR/OV cancer (%)**				0.72
No	33 (37.1)	23 (38.3)	10 (34.5)	
Yes	56 (62.9)	37 (61.7)	19 (65.5)	
**Ethnicity**				0.07
African	1 (1.0)	1 (1.7)	0 (0)	
Ashkenazi Jewish	7 (7.9)	6 (10.0)	1 (3.4)	
Asian	8 (9.0)	7 (11.6)	1 (3.4)	
Caucasian	59 (66.2)	36 (60)	23 (79.6)	
East Indian	4 (4.5)	3 (5.0)	1 (3.4)	
Hispanic	2 (2.2)	1 (1.7)	1 (3.4)	
Middle Eastern	3 (3.5)	2 (3.3)	1 (3.4)	
Mixed	1 (1.1)	1 (1.7)	0 (0)	
West Indies	2 (2.2)	1 (1.7)	1 (3.4)	
Missing/Unknown	2 (2.2)	2 (3.3)	0 (0)	
**Knowledge Score**				0.34
Median (Min,Max)	8 (2,11)	9 (2,11)	8 (3,11)	
Mean (sd)	8.1 (2.0)	8.2 (2.0)	7.9 (2.0)	
**MICRA Total Score**				0.53
Median (Min,Max)	15 (0,75)	15 (0,71)	16 (0,75)	
Mean (sd)	18 (15)	16.9 (13.6)	20.3 (17.6)	
**Distress subscale**				0.58
Median (Min,Max)	0 (0,30)	0 (0,22)	0 (0,30)	
Mean (sd)	2.9 (5.7)	2.3 (4.5)	4.1 (7.5)	
**Uncertainty subscale**				0.85
Median (Min,Max)	6 (0,35)	6 (0,35)	6 (0,34)	
Mean (sd)	8.9 (8.6)	8.4 (7.8)	9.9 (10.1)	
**Positive Experience subscale**				0.51
Median (Min,Max)	15 (0,20)	16 (0,20)	13 (5,20)	
Mean (sd)	14.1 (5.5)	14.3 (5.6)	13.7 (5.3)	
**Correct recall of genetic test result[b] (%)**	86 (97.7)	59 (98.3)	27 (96.4)	0.54
**Discussed result with a relative**	84 (94.4)	57 (95.0)	27 (93.1)	0.66

[a] Response missing for 1 GMT participant; [b] Response missing for 1 OMT participant

Table S5. Multivariable analysis for changes in knowledge scores.

Covariate	β	95% CI	*p*-value
**Group**			**0.035**
GMT	Reference		
OMT	0.95	0.06,1.83	
**Diagnosis**			0.57
Breast	Reference		
Ovarian	−0.27	−1.21, 0.67	
**Age at diagnosis**	−0.03	−0.07, 0.008	0.12
**Family history**			0.85
No	Reference		
Yes	0.008	−0.79, 0.96	

Total sample size of *n* = 89

Table S6. Univariable analysis of MICRA questionnaire.

Covariate	Total		Distress		Uncertainty		Positive Experience	
	**β (95% CI)**	***p*-value**	**β (95% CI)**	***p*-value**	**β (95% CI)**	***p*-value**	**β (95% CI)**	***p*-value**
**Group**		0.33		0.15		0.39		0.59
GMT	Reference		Reference		Reference		Reference	
OMT	3.33 (−3.33, 9.99)		1.82 (−0.68, 4.32)		1.67 (−2.14, 5.47)		−0.66 (−3.09, 1.77)	
**Diagnosis**		0.71		0.74		0.61		0.45
Breast	Reference		Reference		Reference		Reference	
Ovarian	1.33 (−5.65, 8.3)		0.44 (−2.19, 3.07)		−1.04 (−5.02,2.94)		−0.98 (−3.51, 1.55)	
**Age at diagnosis**	0.05 (−0.23, 0.33)	0.75	−0.003 (−0.11, 0.1)	0.96	−0.02 (−0.18, 0.14)	0.81	−0.05 (−0.16, 1.05)	0.29
**Family History**		0.47		0.82		0.87		**0.02**
No	Reference		Reference		Reference		Reference	
Yes	2.37 (−4.11, 8.84)		0.29 (−2.17, 2.74)		0.32 (−3.4, 4.03)		−2.72 (−5.01, −0.44)	

Total sample size of n=89

Table S7. Modified PAHC Tool.

**You have been offered genetic testing because of your diagnosis of cancer. Please answer the following quesiton about your thoughts on genetic testing by placing a box in the appropriate box below[a]:**	
**Domain & Items**	**Cronbach's α**
**ereditary Predisposition Domain**	**0.88**
I am worried about the chance of finding out that I am a carrier of a genetic mutation.	
I am worried about having to make a decision about possible risk reducing options (e.g. preventive surgery or high risk screening).	
I am worried about how I will cope with my genetic test results.	
**ractical Issues Domain**	**0.69**

I am worried about how my genetic test results will impact my daily life (at home, at work, at school, or with hobbies).	
I am worried about how my genetic test results will impact my ability to obtain life insurance.	
**amily Problems Domain**	**0.60**
I feel misunderstood by my family and/or friends regarding my decision to have genetic testing.	
I am bothered by the lack of support regarding my decision to have genetic testing from my family and/or friends.	
I am worried about my family's ability to function because of my decision to have genetic testing.	
I feel genetic testing may benefit my family members. [not scored]	
I am worried about contacting family members about my genetic test results.	
I feel burdened by a sense of responsibility towards my family about my decision to have genetic testing.	
**Living with Cancer Domain**	**0.76**
I am burdened by my cancer diagnosis or cancer treatment.	
I am worried about the chance of getting a **new** diagnosis of cancer.	
I feel that genetic testing can help me understand why I developed cancer. [not scored]	
I am worried about the chance that my family members will get cancer.	
**eneral Emotions Domain**	**0.89**
I feel anxious.	
I feel insecure about the future.	
I have questions about life and death.	
I feel depressed.	
I feel glad that I am having genetic testing. [not scored]	
**hild-Related Issues Domain**	**0.77**
you have children, please answer the following questions:	
I feel guilty about the chance of passing on a genetic mutation to my children.	
I am worried about telling my children about my genetic test results.	
I am worried about the chance that my children may develop cancer.	

[a] Participants were provided with 'Not at all', 'A Little', 'Quite a Bit' or 'Very Much', which were coded as 1, 2, 3, or 4, respectively.
